# An assessment of knowledge and attitude toward antibiotic misuse by small-scale broiler farmers in Bogor, West Java, Indonesia

**DOI:** 10.14202/vetworld.2022.707-713

**Published:** 2022-03-25

**Authors:** Rusman Efendi, Etih Sudarnika, I. Wayan Teguh Wibawan, Trioso Purnawarman

**Affiliations:** 1 Department of Public Health, Faculty of Public Health, Universitas Muhammadiyah Jakarta, South Tangerang City 15419 Indonesia; 2 Department of Animal Disease and Veterinary Public Health, Faculty of Veterinary Medicine, Institut Pertanian Bogor, Bogor 16680, Indonesia

**Keywords:** antibiotic resistance, attitude, broilers, knowledge, use of antibiotics

## Abstract

**Background and Aim:**

Antibiotics are often overused and misused by broiler farmers. Moreover, this practice may lead to antibiotic resistance. Antibiotics may be used for various purposes such as therapy, prophylaxis, flushing, and growth promoters. The study aimed to examine the association of knowledge and attitudes with antibiotics used by broiler farmers.

**Materials and Methods:**

The study design was cross-sectional. The data were obtained from interviewing 132 farmers’ households in Bogor District, West Java, Indonesia. The outcome variable was antibiotic use, whereas the independent variables included knowledge and attitude toward antibiotic resistance. The statistical analysis used a t-test and correlation test.

**Results:**

A total of 78% of broilers farmers use antibiotics, and most of the farmers used antibiotics for flushing and prophylaxis. Furthermore, antibiotic use was associated with broiler farmers’ knowledge and attitudes toward antibiotic resistance. However, there is no significant correlation between the duration of antibiotics use and their knowledge and attitude.

**Conclusion:**

The use of antibiotics in broilers is still high in Bogor, and most of the used antibiotics belong to the Medically Important Antimicrobial category. In general, the use of antibiotics in broilers is influenced by knowledge.

## Introduction

Antibiotics remain the most widely used medicine to treat infections caused by bacteria. They have effectively prevented millions of deaths each year [1]. Nevertheless, it is often overused and misused, which leads to antimicrobial resistance [2]. The misuse and overuse of antibiotics accelerate the incidence of antibiotic resistance, and it also stems from poor infection prevention and control [3-6]. Furthermore, antibiotic residues in consumed food can interact with the microbiome in the human body, causing bacteria to develop antibiotic resistance, which can persist in the human gut for years [7,8]. For instance, *Enterococci* resistance to erythromycin can persist after 1 year of exposure. Eventually, they can also become resistant to macrolides. *Staphylococcus* resistance to macrolides was detected up to 4 years after using clarithromycin [9].

Antibiotic resistance causes first-line antibiotics to become ineffective in treating infectious diseases. Thus, the duration of illness, hospitalization, and treatment will be longer. In addition, it has a substantial impact on society, economy, family, and community [3,6]. The World Health Organization (WHO) has made various efforts to control antibiotic resistance, including in livestock production. Farmers may only give antibiotics under the supervision of a veterinarian. Hence, they may not provide antibiotics as feed additives or prevent disease, administer vaccines, promote sound farming practices, and apply biosecurity without professional guidance [6,10,11]. Control efforts will be challenging to implement without various data related to antibiotic use. Measuring and analyzing the use of antibiotics are an essential steps toward obtaining veritable sources of information for developing appropriate strategies on antibiotic use. Developed and middle-income countries already have data on antibiotic usage in humans and animals. However, antibiotic use data are often unavailable or are limited in low-income countries [2].

Farmers usually use antibiotics for various purposes such as therapy, prophylaxis, flushing, and growth promoters. Koirala *et al*. [12] found that the prevalence of antibiotic use in livestock was 90%. Approximately 22% of farms used antibiotics as prophylaxis, whereas 78% used them for therapy. In addition, providing antibiotics to healthy broilers aged 1-3 d is common among farmers in Indonesia. The purpose is to prevent infection from pathogenic bacteria, known as flushing [13].

Furthermore, the government has prohibited antibiotics used for growth promoters, whereas for therapeutical purposes must be under the supervision of a veterinarian [14]. Profit is the primary motive of farmers irrationally used antibiotics [15]. Farmers who use antibiotics can generate more revenue because antibiotics can maintain health and increase broiler growth [16-18]. Moreover, the irrational use of antibiotics was influenced by various factors, including lack of knowledge and attitude toward the overuse of antibiotics and their effect on human health [17]. The Minister of Agriculture, Republic of Indonesia, has issued ministerial regulation number 14 in 2017. This regulation prohibits the use of antibiotics as growth promoters and limits their use for therapeutics [14]. In 2019, the Minister of Agriculture, Republic of Indonesia, also issued a circular banning colistin as a veterinary drug [19]. Colistin is in the polymyxin class of antibiotics. It is the last resort option of drugs that are usually used to treat patients with multidrug resistance. However, it is frequently used in broiler farming as prophylaxis. A study of 47 farms using colistin in Bogor found *Escherichia coli* resistant to colistin by 8.51% [20].

The existence of these regulations should have an impact on the use of antibiotics in broiler farmers, but not much was known about the impact of these changes. This study aimed to investigate the current situation of antibiotics used in broiler farming in Indonesia and its association with the knowledge and attitude of the farmers.

## Materials and Methods

### Ethical approval

Ethical approval and informed consent: The proposal of this study was approved by Human Research Ethics Committee of the Bogor Agricultural University, with number: 339/IT3.KEPMSM-IPB/SK/2021. Further, informed consent was obtained from all the participants after explaining the aim of the study in clear language.

### Study period and location

The study was conducted from March to July 2021 in the western, northern, southern, and central areas of Bogor District, West Java, Indonesia. This area is mostly highlands, hills, and mountains. Bogor District is close to the National Capital, so it is an area that has strategic potential in the development and growth of the economy and services.

### Study design

The study was cross-sectional. In this study, the data on the knowledge, attitudes, and behavior of antibiotic use variables consisting of the use of antibiotics, the use of combinations of antibiotics, the duration of antibiotic use, the use of antibiotics as flushing, the use of combinations of antibiotics as flushing, the duration of the use of antibiotics as flushing, the use of antibiotics as prophylaxis, the use of antibiotics combination of antibiotics as prophylaxis, duration of use of antibiotics as prophylaxis, use of antibiotics as therapy, use of herbs, and use of probiotics were taken in the same period.

### Population and sampling

The unit of study was farmer household level, and the behavior interview was conducted with one respondent from each farmer household. We selected broiler farming in the Bogor District, West Java. It has the largest number of broiler farming in Indonesia, which is approximately 15,41,27450 of the total population of Indonesia of 2,970,493,660 broilers and approximately 1321 farmer households. The sampling size was calculated using the Lemeshow formula [21]. The minimum sample size was 96. This number was based on the assumption of 5% alpha and absolute precision 10%. The sampling technique was drawn by purposive sampling. Ultimately, the sample size consists of 132 farmers’ households in western, northern, southern, and central regions. The sample inclusion criteria were small-scale broiler farmers of the number of broilers 5001-50000, raising livestock in the past 3 months. Exclusion criteria involved farmers who were raising livestock for the 1^st^ time.

### Data collection

Data were taken using a questionnaire through interviews. The questionnaire was tested for validity and reliability to ensure its feasibility before being used. All data on antibiotic use behavior were obtained using closed questions with yes or no answer choices, except for data on the duration of antibiotic use, which was obtained from respondents by mentioning the length of time they used them. Knowledge of antibiotic use and resistance data was measured using 11 closed questions, with each question having a weighted value of 9.1. The lowest total knowledge value of respondents was 0, and the highest was 100. Eleven attitude statements with four scales measured attitudes toward antibiotic use. The scale values for positive statements are as follows: Strongly disagree=1, disagree=2, agree=3, and strongly agree=4. The scale value for negative statements includes strongly disagree=4, disagree=3, agree=2, and strongly agree=1. The total score of the lowest respondent’s attitude is 11, and the highest is 44.

### Statistical analysis

Data analysis was conducted using a t-test and correlation test. The t-test was conducted to ascertain the difference in mean knowledge and attitude concerning antibiotic resistance and the use of antibiotics. Furthermore, the use of combinations of antibiotics, the use of antibiotics for flushing, the use of combinations of antibiotics for flushing, the use of antibiotics as prophylaxis, the use of combinations of antibiotics as prophylaxis, the use of antibiotics as therapy, the use of herbs, and the use of probiotics were evaluated. A correlation test was conducted to examine the relationship between farmers’ knowledge and attitudes about the use and resistance of antibiotics, the duration of antibiotic use, the duration of antibiotics use for flushing, and the duration of antibiotic use for prophylaxis. The strength of the relationship in the correlation test was measured regarding the correlation coefficient value. The following criteria indicated the correlation strength: a very weak correlation strength level=0.00-0.25, sufficient=0.26-0.50, strong=0.51-0.75, very strong=0.76-0.99, and perfect=1.00 [22]. This study uses a significant level (a) of 5%.

## Results

The area of Bogor Regency is 2663.81 km^2^. Bogor Regency has the highest broiler population in West Java and the broiler population in West Java is the largest in Indonesia. Broiler farms are spread over 36 subdistricts out of 40 subdistricts in the district.

Broiler farmers use as much as 78% of antibiotics. Antibiotics that farmers use widely include amoxicillin, enrofloxacin, and colistin. Based on the antimicrobial class, most of the antibiotics used by broiler farmers, including the Penicillin (aminopenicillin) and Quinolones classes, accounted for 33.3% each. Both, according to the WHO [23], are included in the critically important category in the Medically Important Antimicrobials (Table-1) and include a World Organization for Animal Health list of antimicrobial agents of veterinary importance [24].

**Table-1:** Use of antibiotics by farmers in Bogor by name, class, and category of antibiotics.

Antimicrobial agents	n (%)	Antimicrobial class	n (%)	Medically important antimicrobials categorize
Amoxicillin	43 (32.6)	Penicillins (aminopenicillins)	44 (33.3)	Critically important
Ampicillin	1 (0.8)			
Lincomycin	1 (0.8)	Lincosamides	1 (0.8)	Highly Important
Erythromycin	11 (8.3)	Macrolides	22 (16.7)	Critically Important
Tylosin	11 (8.3)			
Colistin	22 (16.7)	Polymyxins	22 (16.7)	Critically Important
Enrofloxacin	35 (26.5)	Quinolones	44 (33.3)	Critically Important
Levofloxacin	1 (0.8)			
Ciprofloxacin	5 (3.8)			
Norfloxacin	3 (2.3)			
Tetracycline	1 (0.8)	Tetracyclines	23 (17.4)	Highly Important
Doxycycline	12 (9.1)			
Oxytetracycline	10 (7.6)			
Sulfadiazine	13 (9.8)	Sulfonamides	26 (19.7)	Highly Important
Trimethoprim	13 (9.8)			
No antibiotics	29 (22.0)	No antibiotics	29 (22.0)	-

The farmers’ behavior in using drugs to control disease and increase growth were grouped into those who use antibiotics, herbs, and probiotics. The statistical test results showed that Farmers who used antibiotics had good knowledge about antibiotics and resistance than those who did not (p<0.05). The farmers who used herbs had a better attitude towards antibiotics than those who don’t use them (p<0.05). There was no significant difference in knowledge and attitude of farmers using probiotics (Table-2).

**Table-2:** The results of the t-test for differences of knowledge and attitudes based on the use of antibiotics, herbs, and probiotics.

Variables	n (%)	Knowledge			Attitude		
	
		Mean	p-value	CI (95%)	Mean	p-value	CI (95%)
Use of antibiotics							
Yes	103 (78.0)	71.4	0.000**	2.57-7.27	51.3	0.279	−1.49-5.11
No	29 (22.0)	66.5			49.5		
Use of herbs							
Yes	75 (56.8)	70.6	0.481	−1.30-2.75	52.7	0.004**	1.29-6.64
No	57 (43.2)	69.9			48.7		
Use of probiotics							
Yes	9 (6.8)	72.2	0.337	−2.50-6.48	52.7	0.486	−5.54-9.39
No	123 (93.3)	70.2			50.8		

*Significant at p*≤*0.05

** Significant at p*≤*0.01, CI=Confidence interval

For disease prevention in broilers, our study found that farmers who use herbs for growth and animal health were approximately 56.8%. This number is still much higher than those who use probiotics, but lower than those who use antibiotics. The lack of herbs used may be due to insufficient knowledge on the benefit of herbs and few standardized herbal medicines for animals. It could also be due to the lack of socialization on herbal research for animal growth and health. Although knowledge of herbs is still lacking, the attitude of farmers shows a positive attitude on the use of herbs in broilers (Table-2).

The use of antibiotics in livestock in broilers is divided into several ways. Antibiotic use may involve a combination and a single antibiotic, and it is often used for flushing, prophylaxis, and therapy. Farmers who used antibiotics for flushing also used combinations of antibiotics. The same behavior also occurs in farmers who use antibiotics as prophylaxis. Regarding the level of knowledge about antibiotics and resistance, all the farmers, both those who used antibiotics as flushing and prophylactics, had better knowledge than those who did not. Likewise, farmers who use a combination of various antibiotics such as flushing, and prophylaxis also had good knowledge of antibiotics and resistance than those who use a single antibiotic (Table-3).

**Table-3:** Results of t-test for difference of knowledge and attitudes based on the use of antibiotics.

Variables	n (%)	Knowledge			Attitude		
	
		Mean	p-value	CI (95%)	Mean	p-value	CI (95%)
Use of antibiotic combinations							
Yes	60 (45.5)	72.0	0.003**	1.11-5.13	50.4	0.456	−3.79-1.71
No	75 (54.5)	68.9			51.4		
Use of antibiotics as a flushing							
Yes	99 (75.0)	71.4	0.001**	1.77-6.34	51,3	0.315	−1.54-4.77
No	33 (25)	67.3			49.7		
Use of a combination of antibiotics as a flushing							
Yes	51 (38.6)	72.0	0.007**	0.77-4.69	50.6	0.692	−3.38-2.25
No	81 (61.4)	69.3			51.2		
Use of antibiotics as prophylaxis							
Yes	74 (56.1)	71.3	0.030*	0.22-4.32	51.8	0.155	−0.76-4.73
No	58 (43.1)	69.1			49.8		
Use of combination antibiotics as prophylaxis							
Yes	42 (31.8)	72.7	0.001**	1.40-5.68	51.7	0.431	−1.76-4.11
No	90 (68.2)	69.2			50.6		
Use of antibiotics as therapy							
Yes	11 (8.3)	68.3	0.255	−5.88-1.57	48.1	0.223	−8.00-1.88
No	121 (91.7)	70.5			51.2		

* Significant at p-value ≤ a 0.05

** Significant at p-value ≤ a 0.01, CI=Confidence Interval

Knowledge of antibiotic use and resistance was significantly correlated positively with the duration of use of antibiotics as flushing (p<0.05). The strength of the correlation is at a sufficient level with a coefficient value of 0.305 (Table-4).

**Table-4:** The results of the correlation test for the duration of antibiotic use with knowledge and attitudes.

Variables		Correlation coefficient	p-value	Correlation level
Knowledge	Duration of use of antibiotics	0.167	0.056	No correlation
	Duration of use of antibiotics as flushing	0.305	0.000**	Moderate
	Duration of use of antibiotics as prophylaxis	0.054	0.539	No correlation
Attitude	Duration of use of antibiotics	0.122	0.165	No correlation
	Duration of use of antibiotics as flushing	0.164	0.060	No correlation
	Duration of use of antibiotics as prophylaxis	0.112	0.199	No correlation

*Significant at p*≤*0.05

** Significant at p*≤*0.01

## Discussion

### Use of antibiotics in broilers

The study found that antibiotic use in broiler farming is considerably high in Bogor District. Various antibiotics are administered to healthy chicken broilers, including those in the critically important antimicrobial class. The overuse of these types of antibiotics would complicate the treatment of bacterial infection because of resistance. In addition, the broiler farmers widely used colistin, which is banned in many countries. Colistin has long been considered a drug of last resort for treating gastrointestinal infections and bacteremia caused by multidrug-resistant bacteria in humans. In 2019, the Indonesian government banned colistin use in animals to prevent antimicrobial-resistant in humans [19]. Likewise, China has banned colistin as a feed additive for animals since 2015 [25]. Moreover, Japan has changed the rules for the therapeutic use of colistin from a drug of the first choice to a second choice. They also banned the sale of colistin as a feed additive in animals in 2018 [26].

Knowledge and attitudes of farmers may influence the overuse of critically important antibiotics. Most of the farmers understand the effect of antibiotic misuse on public health. However, they noticed that antibiotics had plenty of benefits for the health and growth of broilers. Therefore, farmers tend to change the pattern of using antibiotics from feed to drinking water additives, although the effectiveness of this method is not yet known. The use of antibiotics in farmers who do not comply with the indicated regulations is likely to occur [27]. Nonenforcement of laws regulating antimicrobial usage, weak financial status, low education and expertise, and nomadic culture influence the misuse of antimicrobial in livestock [28]. A study by Widiasih *et al*. [29] and Furi *et al*. [30] found antibiotic residues in chicken meat after the ministerial regulation. The implementation of the regulation needs to be enforced by educating the farmers about the adverse effects of antibiotic abuse on human, animal, and environmental health.

### Knowledge, attitude, and antibiotic use in broilers

Farmers make considerable efforts to promote growth and maintain the health of broilers. In addition to antibiotics, they have safer alternatives using herbs and probiotics. However, antibiotics used for growth promoters and drugs in animals remain high. The farmers may not clearly understand the benefits and dangers of antibiotics. Prior research revealed that broiler farmers have insufficient knowledge about antibiotics [31-33]. Another study found that farmers’ knowledge about antibiotic resistance is still limited. Hence, farmers consider that antibiotic use to increase chicken productivity and growth still plays an important role [34-36]. The existence of socialization of the prohibition encourages farmers to discover more about drugs that can be used. This allows farmers to get erroneous knowledge or misuse their knowledge about antibiotics used in broilers.

In practice, most farmers still use antibiotics for broiler growth and health because they are considered more effective. However, the combination of antibiotics is effective in treating, preventing, and maintaining the balance of normal flora in the digestive tract of broilers. For example, a study found that using a combination of antibiotics in treatment provided an excellent response to treat *E. coli* infection in the chicken broiler [37,38]. In addition, the combination of tylosin and gentamicin effectively balances the normal flora in the duodenum, making chicken growth optimal [39].

We found that farmers who use antibiotics in broilers for flushing and prophylaxis have good knowledge about antibiotic use and resistance. This indicates the misuse of antibiotics because, generally, farmers know that antibiotics cannot freely be used in livestock. The high usage may be due to farmers wanting to get the benefits of antibiotics for growth and control infection without paying attention to the long-term effects of antibiotic resistance and other hazards. For example, Nonga *et al*. [40] found that 90% of the respondents knew of the antimicrobial withdrawal period. Their results indicated that 90% of the respondents knew of the antimicrobial withdrawal period. However, 95% of farmers slaughter their chickens before the recall period for fear of death and are unaware of the effects of antimicrobial residues on humans.

Understanding the benefits of antibiotics on animal growth and health is not accompanied by a sufficient understanding of the dangers of antibiotics. The use of antibiotics as drugs or feed additives can increase livestock productivity, which would generate profits for farmers [41]. According to Cardinal *et al*. [17], broilers with antibiotics in their feed had higher body weight gain and better feed conversion than those not fed antibiotics on a diet. The discontinuation of antibiotic feeds additives could increase production costs [42]. From the results of this study, it was found that farmers’ knowledge of the harmful effects of antibiotics on humans was 17.4%.

In comparison, the knowledge on the dangers to animals and the dangers to the environment amounted were 4.5% and 6.8%, respectively (Table-5). A similar situation was found by Khan *et al*. [43] that as many as 77.5% of live chicken traders in the market used antibiotics to maintain chickens’ health while they were in shelters. Approximately 57.5% of live broiler traders know antibiotics as growth promoters. However, only 7.5% of sellers understand the term antibiotic resistance, and 12% have little knowledge of the dangers to human health after the indiscriminate use of antibiotics. Therefore, farmers should be adequately educated on indiscriminate antibiotic use’s dangers and adverse impacts. This should be accompanied by the socialization of regulations limiting or prohibiting antibiotics in broilers.

**Table-5:** Knowledge of respondents about the use and resistance of antibiotics in broilers.

Knowledge Items	Correct answer n (%)
Antibiotics should not be used as growth promoters	37 (28)
Antibiotics should not be used as prophylaxis	4 (3)
Antibiotics may be used for treatment under veterinary supervision	16 (12.1)
Using antibiotics during farming can harm human health	23 (17.4)
Using antibiotics during farming can harm animal health	6 (4.5)
Using antibiotics during farming can harm environmental health	9 (6.8)
Antibiotic resistance can be caused by improper use of antibiotics	101 (76.5)
The use of antibiotics should not be until the time the broiler will be slaughtered	118 (89.4)
The use of antibiotics must be based on the advice of a veterinarian or according to the instructions on the drug labels	120 (90.9)
Selection of antibiotics for treatment should be based on the advice of a veterinarian	67 (50.8)
The withdrawal time of antibiotic use in broilers must be according to veterinary advice or drug labels	122 (92.4)

Among the several variables measuring the duration of antibiotics in broilers, only one variable correlated with knowledge. In addition, this includes the duration of using antibiotics for flushing. Broiler farmers have long used antibiotics in newborn broiler chicks entering the cage at 1-5 days old. Farmers know that flushing using antibiotics is quite effective in controlling disease in broilers. The duration of antibiotic use is critical because, according to some studies, *E. coli* resistance resulted from prolonged antibiotic treatment [44,45].

## Conclusion

Farmers who use antibiotics in broilers were approximately 78%. Most of the antibiotics used are classified as a critically important antimicrobial category. On average, farmers who use antibiotics have good knowledge about the use and resistance of antibiotics than those who do not use antibiotics. Farmers who use herbs have a better attitude toward antibiotic resistance than those who do not use herbs. Farmers who employed the use of combinations of antibiotics, the use of antibiotics as flushing, the use of combinations of antibiotics as flushing, the use of antibiotics as prophylaxis, and the use of combinations of antibiotics as prophylaxis had good knowledge about antibiotic use and resistance than those who did not use them. Knowledge of antibiotic use and resistance correlated with the use duration of antibiotics for flushing.

## Authors’ Contributions

RE, ES, WT, and TP: Conceived the idea. RE and ES: Designed the study, developed the theory and prepared the tools and materials. RE: Collected the data. RE and ES: Analyzed the data. RE: Wrote the manuscript with input from all authors. All authors discussed the results and contributed to the final manuscript, provided critical feedback, and helped shape the research, analysis, and manuscript. All authors read and approved the final manuscript.
